# Implementation of patient portals: the perspective of the professional care provider

**Published:** 2012-06-15

**Authors:** Wynand J.G Ros, Karin ter Horst

**Affiliations:** Julius Centre, UMC Utrecht, The Netherlands; Julius Centre, UMC Utrecht, The Netherlands

**Keywords:** e-health, patient portal, nurse, usability, implementation

## Abstract

**Introduction:**

A few hospitals in the Netherlands have been introducing digital patient portals. Patient portals are secured internet environments for patients to log into from their homes to exchange information with their care provider. Such portals can be seen as an application of e-health that encourages patient’s self management. User acceptance is an important precondition for successful implementation. Research on patient user acceptance has already occurred. This project is about the user acceptance of the professional healthcare provider. The focus is on nurses, because in the Netherlands nurses play a crucial role in developing and implementing a portal, as well as in introducing the portal to the patient. Besides that, they have to integrate the portal tasks with their other work.

**Aim:**

This qualitative research describes the extent to which and the manner in which nurses accept digital patient portals. It is investigated how the portals are developed and implemented, how nurses use and appreciate the portals and which factors are important for realizing user acceptance of professional health care providers.

**Method:**

In-depth interviews were executed with 18 respondents of two University Medical Centers in the Netherlands. The group consisted of 12 nurses and nursing specialists, 2 doctors, 2 researchers, 1 communication officer and 1 manager. Interview topics were derived from the Technology Acceptance Model (TAM) and the Innovation Diffusion Theory (IDT).

**Results:**

The results show that the degree of acceptance among nurses is great. Nurses see the introduction of patient portals as an inevitable development. Essential for nurses’ acceptance is the ‘perceived usefulness’, especially from patient’s perspective. Perceived usefulness from their own perspective (ease-to-use) also plays a role but this is of less importance. The e-consult is relevant. However, it will not substitute usual (face-to-face or telephone) contacts, unless e-consult is part of the scheduled treatment plan. But this is still rarely the case and actually a cultural change in professional practice, a digital revolution is needed. Moreover, the work is not yet designed for e-health; time and costs for e-health are not yet reimbursed in the Dutch context. Nurses see the portal as an additional service for patients, because it offers them the possibility for asking questions at any time and place suitable for the patient. Some nurses experience an increase in work load, because patients ask more non-urgent questions that otherwise would not be asked. Other nurses observe a decrease of workload, because of a decrease of consultations about laboratory test results.

**Conclusion:**

The user acceptance from nurses is great. Most important motive is the perceived usefulness for the patient. The patient portal is seen as an extra service for the patient, and the nurses are prepared to deliver this service. There is a risk of increased workload. Exploiting the potentials for greater efficiency requires a cultural change in professional practice.

